# Motor cortical plasticity and its correlation with motor symptoms in Parkinson's disease

**DOI:** 10.1016/j.ensci.2022.100422

**Published:** 2022-09-01

**Authors:** Shotaro Moriyasu, Takahiro Shimizu, Makoto Honda, Yoshikazu Ugawa, Ritsuko Hanajima

**Affiliations:** a Division of Neurology, Department of Brain and Neurosciences, Faculty of Medicine, Tottori University, Yonago, Tottori 683-8504, Japan; b Department of Human Neurophysiology, School of Medicine, Fukushima Medical University, Fukushima 960-1295, Japan

**Keywords:** Quadripulse magnetic stimulation (QPS), Parkinson's disease, Primary motor cortex, Bradykinesia, Rigidity, long-term potentiation (LTP), L-DOPA, L-3,4-dihydroxyphenylalanine, MDS-UPDRS, the Movement Disorders Society Unified Parkinson's Disease Rating Scale, MMSE, Mini-Mental State Examination, MoCA-J, the Japanese version of Montreal Cognitive Assessment

## Abstract

**Background:**

The relationship between abnormal cortical plasticity and parkinsonian symptoms remains unclear in Parkinson's disease (PD).

**Objective:**

We studied the relationship between their symptoms and degree of Long-term potentiation (LTP)-like effects induced by quadripulse magnetic stimulation (QPS) over the primary motor cortex, which has a small inter-individual variability in humans.

**Methods:**

Participants were 16 PD patients (drug-naïve or treated with L-DOPA monotherapy) and 13 healthy controls (HC). LTP-like effects by QPS were compared between three conditions (HC､PD with or without L-DOPA). In PD, correlation analyses were performed between clinical scores (MDS-UPDRS, MMSE and MoCA-J) and the degree of LTP-like effects induced by QPS.

**Results:**

In PD, QPS-induced LTP-like effect was reduced and restored by L-DOPA. The degree of the LTP was negatively correlated with MDS-UPDRS Part I and III scores, but not with MMSE and MoCA-J. In the sub-scores, upper limb bradykinesia and rigidity showed a negative correlation with the LTP-like effect whereas the tremor had no correlation.

**Conclusions:**

Our results suggest that motor cortical plasticity relate with mechanisms underlying bradykinesia and rigidity in the upper limb muscles. LTP induced by QPS may be used as an objective marker of parkinsonian symptoms.

## Highlights

• Quadripulse magnetic stimulation (QPS) was applied to early PD patients.
• L-DOPA restored QPS-induced LTP of the primary motor cortex in early PD patients.
• The degree of LTP was negatively correlated with the severity of motor symptoms.
• Upper limb bradykinesia and rigidity had a strong negative correlation with LTP.

## 1 Introduction

The pathophysiological mechanisms underlying parkinsonian symptoms have not been fully elucidated. Synaptic plasticity, such as long-term potentiation and depression (LTP/LTD), has been claimed to partly contribute to the pathophysiology of Parkinson's disease (PD) [1,2]. In PD animal models, LTD/LTP induction in the striatal medial spiny neurons was impaired [1] and restored by dopamine [3]. Impairment of depotentiation was suggested to reflect the generation of dopamine-induced dyskinesia [2]. Given these findings, dopamine-dependent neural plasticity could have some roles in parkinsonian symptoms. However, the restoration of plasticity by L-DOPA and the relationship between the degree of plasticity and clinical symptoms remain unclear in PD patients.

Recently, various non-invasive brain stimulation (NIBS) techniques have been developed to induce motor cortical plasticity in humans. Several studies using paired associative stimulation (PAS) [[4], [5], [6]] or theta burst stimulation (TBS) [7,8] reported impairments of cortical plasticity in PD patients. Impairment of depotentiation in patients with dopamine-induced dyskinesia were also detected using theta burst stimulation (TBS) [9]. However, the relationships between LTP-like effects and parkinsonian symptoms or L-DOPA intake were inconsistent among the previous studies. The reduced LTP-like effects after PAS were restored by L-DOPA intake [4,5], while L-DOPA intake did not influence LTP-like effects after TBS [7]. The relation between LTP-like effects after PAS and parkinsonian symptoms was variable; namely, none [10,11], negative [4,12], and positive [13] correlations were reported. These inconsistent results were possibly due to different mechanisms underlying LTP-induction between the two NIBS methods, large inter-individual variability of the two techniques, different clinical stages of the studied patients, and variable medications [14,15]. We developed another NIBS technique to induce homotopic plasticity of the primary motor cortex (M1), quadripulse magnetic stimulation (QPS) [16], which was shown to have less inter-individual variability than other NIBS procedures [17,18]. In healthy subjects, L-Dopa enhanced LTP-like effects of QPS [19,20].

Here, to clarify whether the homotopic plasticity of the M1 is enhanced by dopamine in PD patients, and whether M1 plasticity relates with parkinsonian symptoms, we studied the LTP-like effects in PD patients (drug-naïve or treated with L-DOPA monotherapy) using QPS and compared those with normal values obtained from age matched healthy controls. We hypothesized that the LTP-like effect induced by QPS could reflect dopamine-dependent neural plasticity and show a correlation with PD symptoms. If so, QPS could be used to estimate parkinsonian involvement objectively.

## 2 Methods

### 2.1 Participants

We studied 16 PD patients (7 males, 9 females; mean age ± 1 standard error of the mean(SEM) = 70.06 ± 2.22 years; range 45–83 years) who were naïve to anti-Parkinsonian medication or taking only L-DOPA. Thirteen healthy controls (HC) (6 males, 7 females; age 72.92 ± 1.71 years; range 65–86 years) were also enrolled in the study. We excluded patients taking D2/3 dopamine agonists, because the half-life time of these drugs is much longer than L-DOPA. All patients were recruited from those visiting the Department of Neurology, Tottori University Hospital. We recruited patients who had clinically established and clinically probable PD in accordance with MDS Clinical Diagnostic Criteria for Parkinson's Disease [21], or clinically established early PD in accordance with the MDS Clinical Criteria for Clinically Established Early Parkinson's Disease [22], and also whose disease duration was shorter than 7 years. The clinical data are shown in Table 1. We excluded patients with alcohol or illegal drug abuse, seizure episodes, or other neurologic or psychiatric disorders. None had contraindication to transcranial magnetic stimulation (TMS) [23]. All the participants were right-handed.

**Table 1:** Clinical features of Parkinson's disease patients.

								MDS-UPDRS Part III	
Patient	Age	Gender	Disease duration (year)	Hoehn & Yahr	L-Dopa dosage (mg/day)	MMSE	MoCAJ	PD without L-DOPA	PD with L-DOPA
1	76	F	1	2	0	29	27	15	15
2	65	F	1	2	200	29	24	10	3
3	65	F	1	2	200	28	28	13	5
4	72	F	1	2	200	29	24	31	20
5	63	M	1	2	300	29	26	15	9
6	72	F	1	2	400	26	24	27	23
7	45	M	2	2	200	30	25	19	17
8	73	F	2	2	300	30	23	26	22
9	70	M	2	2	400	23	26	46	26
10	75	F	2	2	400	27	25	37	20
11	83	F	4	1	200	29	21	31	22
12	67	M	4	2	250	25	25	34	19
13	83	F	5	2	200	29	26	26	16
14	66	M	5	1	300	29	26	31	17
15	72	M	5	2	500	27	26	42	31
16	74	M	7	2	400	27	22	46	35
Mean ± SEM	70.06 ± 2.22		2.75 ± 0.49	1.87 ± 0.09	278.13 ± 30.61	27.88 ± 0.48	24.88 ± 0.46	28.06 ± 2.86	18.75 ± 2.11

F, female; M, male; MMSE, Mini-Mental State Examination; MoCAJ, Japanese version of Montreal Cognitive Assessment.

All the participants provided written informed consent to participate in this study. This study was performed according to the Declaration of Helsinki; the protocol was approved by the Medical Ethics Committee of the Tottori University (No. 17B033).

### 2.2 Clinical measures

Clinical severity of parkinsonian symptoms was assessed with the Movement Disorders Society Unified Parkinson's Disease Rating Scale (MDS-UPDRS) [24]. Among MDS-UPDRS scores, for a detailed assessment of the relationship between clinical symptoms and QPS-induced motor cortical excitability, we extracted the following upper limb scores on the side of the electromyogram (EMG) recorded muscle; “upper limb rigidity on the recorded side” (item 3.3b or 3.3c; scores for recorded side of rigidity in upper extremities), “upper limb bradykinesia on the recorded side” (item 3.4a-3.6a or 3.4b-3.6b; total sum scores of finger tapping, hand movements, and pronation/supination for the recorded side), and “upper limb tremor on the recorded side” (item 3.15a-3.17a or 3.15b-3.17b; total sum scores of postural tremor, kinetic tremor, rest tremor amplitude of upper extremity for the recorded side).

Cognitive function was assessed with Mini-Mental State Examination (MMSE) [25,26] and the Japanese version of Montreal Cognitive Assessment (MoCA-J) [27,28].

### 2.3 EMG recordings

Participants were seated on a comfortable chair during the experiment. EMG activity was recorded from the relaxed first dorsal interosseous (FDI) muscle using surface electrodes placed with a belly-tendon montage. PD patients were recorded on the more affected side. In nine patients who were unable to relax their FDI due to severe tremor, we recorded an EMG from the FDI on the less affected side. We tested the more affected side in seven patients and the less affected side in nine patients. The right side was used in all HCs because they are righthanded. Responses were inputted to an amplifier (BA-1008, TEAC Co. Ltd., Japan); the low-pass filter was set at 3 kHz and the time constant at 0.01 s. Signals were digitized at 20 kHz and stored in a computer for later off-line analyses (MultiStim tracer; Medical Try System, Japan).

### 2.4 Transcranial magnetic stimulation

We delivered a single pulse TMS over the hotspot in the primary motor cortex (M1) for the contralateral FDI with a figure-of-eight magnetic coil (70 mm wing diameter; the Magstim Co. Ltd., UK) connected to a magnetic stimulator (Magstim200; the Magstim Co. Ltd., UK). The coil was held to induce a current in the posterolateral to anteromedial direction in the brain. The hotspot in the M1 was identified as the point where the largest motor-evoked potential (MEP) was elicited. At this point, we measured the active motor threshold (AMT) and resting motor threshold (RMT). The AMT was defined as the intensity sufficient to elicit at least 100 μV MEPs from the FDI in half of the trials when the subjects maintained 5% maximal voluntary contraction, and the RMT as the minimum stimulator intensity eliciting at least 50 μV MEPs in half of the trials in the relaxed target muscle [29]. Each stimulus intensity is shown as percent of the maximum stimulator output (%MSO).

### 2.5 Quadripulse stimulation

**Fig. 1:**
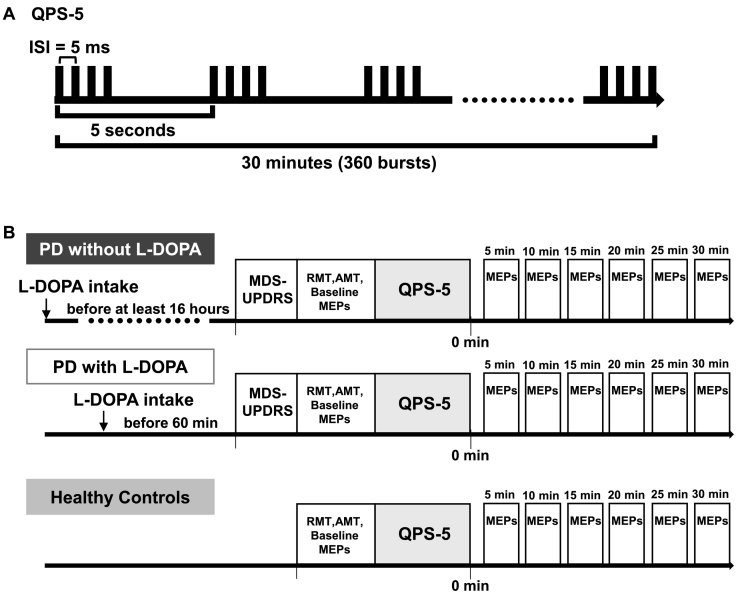
Experimental procedures. (A) The protocol of quadripulse stimulation (QPS). The inter-stimulus interval (ISI) used in this study was 5 ms. (B) Timelines of the experiments. Patients were studied in two separate sessions; “PD with L-DOPA” and “PD without L-DOPA ” states. In “PD without L-DOPA”, patients did not take L-DOPA for at least 16 h, and in “PD with L-DOPA”, they took L-DOPA 60 min before the study. For each session, we assessed MDS-UPDRS, the motor thresholds (RMT/AMT) and 20 baseline motor evoked potentials (MEPs). Immediately after the baseline MEP recordings, we delivered QPS5 for 30 min. After QPS, 20 MEPs were measured every 5 min up to 30 min (5, 10, 15, 20, 25, and 30 min) using the same intensity as the baseline recording. HC were studied only once by the same protocol as that used for PD patients.

We delivered QPS through a combining module (The Magstim Co. Ltd.) connected with four monophasic stimulators (Magstim 200^2^, The Magstim Co. Ltd.). The protocol of QPS is shown in Fig. 1. QPS consisted of bursts of four monophasic TMS pulses repeated every 5 s for 30 min (360 bursts, 1440 pulses). In the present study, we used inter-stimulus intervals of 5 ms (QPS5), which has been reported to be the best interval for LTP induction [16]. QPS was given over the M1 contralateral to the target FDI muscle with a hand-held figure-of-eight coil. The stimulus intensity of QPS was 90% of the AMT for the target FDI muscle.

### 2.6 Study design

The timelines of experiments are shown in Fig. 1. The PD patients were studied on two separate days, once under the condition without taking L-DOPA (“PD without L-DOPA”) and the other while taking L-DOPA (“PD with L-DOPA”). The interval between the two visits were at least one week. In “PD with L-DOPA”, the patient took L-DOPA approximately 60 min before the experiment after the meal. The dose of L-DOPA was the maximum of usual single dose or 100 mg in de novo PD patients (100 mg: 13 patients, 150 mg: 1 patient, 200 mg: 2 patients). In “PD without L-DOPA”, drug-naïve patients visited our hospital without taking drugs, and patients already under L-DOPA medication visited after overnight withdrawal of L-DOPA treatment (at least 16 h). HCs were studied only once and without taking L-DOPA.

For each experimental session in PD patients, we assessed clinical measures, the baseline motor thresholds (RMT/AMT), and 20 baseline motor evoked potentials (MEPs). With single pulse TMS, intensity was set to elicit MEPs of about 0.5 mV in the relaxed FDI. Immediately after the baseline MEP recordings, we delivered QPS5 for 30 min. After QPS, 20 MEPs were measured every 5 min up to 30 min (5, 10, 15, 20, 25, and 30 min) using the same intensity as the baseline recording. We assessed the HCs with the same protocol as that used in PD patients without the evaluation of clinical symptoms.

### 2.7 Data analysis and statistical assessment

We compared age and gender distributions between PD patients and HC using Wilcoxon *t-*test and Pearson's chi-squared test. In PD patients, using paired t-test, we compared MDS-UPDRS Part I, II, III, “upper limb rigidity on the recorded side”, “upper limb bradykinesia on the recorded side”, and “upper limb tremor on the recorded side” between “PD without L-Dopa” and “PD with L-Dopa”. For RMT and AMT, one-way repeated measures analysis of variance (ANOVA) was performed with GROUP (three levels: “PD without/with L-DOPA” and HC) as between-subjects factor.

MEP amplitudes were measured and averaged over each time point. The effects of QPS were assessed by the MEP size ratio, which was defined as mean MEP amplitude at each time point divided by the mean baseline MEP amplitude. For each participants, the average MEP size ratio throughout 5–30 min after QPS was calculated as the grand average MEP size ratio, in order to obtain one value representing the degree of LTP as a whole.

In the analysis of LTP effects, we used two-way repeated measures ANOVA within-subject factors “GROUP” (three levels: “PD without/with L-DOPA” and HC) and “TIME” (six points: 5, 10, 15, 20, 25, and 30 min). For conditions with a significant F-value, we evaluated differences between groups by using post hoc Turkey HSD test.

To study the relationship between clinical symptoms and QPS-induced motor cortical LTP, we studied the correlation coefficients between age, disease duration, L-DOPA dosage, MMSE, MoCA-J, MDS-UPDRS Part I, II, and III scores, “upper limb rigidity on the recorded side”, “upper limb bradykinesia on the recorded side” or “upper limb tremor on the recorded side”, and the grand average of the MEP size ratio using Pearson's product moment correlation coefficient.

All statistical analyses were performed using SPSS software (version 25.0 for Windows; IBM Co. Ltd., New York, USA). For all analyses, *P* value < 0.05 was set as statistically significant. Data were given as mean ± SEM unless otherwise stated.

## 3 Results

### 3.1 Clinical measures

The baseline clinical features are shown in Table 1. Most patients were elderly (older than 65 years old) and in the early stage of PD. No patients showed dyskinesia. No significant differences were found in age (*p* = 0.334) or gender distributions (*p* = 0.897) between PD patients and HC.

**Fig. 2:**
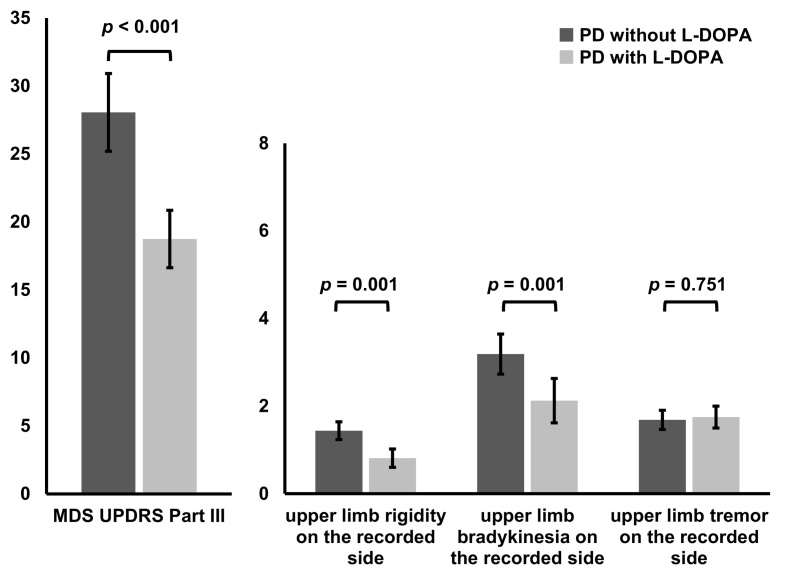
Effects of L-DOPA on motor symptoms. Comparison of MDS-UPDRS Part III scores and its subscores (“upper limb rigidity on the recorded side”, “upper limb bradykinesia on the recorded side” and “upper limb tremor on the recorded side”) between “PD without L-DOPA” (dark grey bars) and “PD with L-DOPA” (light grey bars). The y-axis indicates MDS-UPDRS score. Paired *t*-test showed significant differences in MDS-UPDRS Part III score, “upper limb rigidity on the recorded side” and “upper limb bradykinesia on the recorded side”.

The MDS-UPDRS Part III score was lower “PD with L-DOPA” compared to “PD without L-DOPA” (PD without L-DOPA vs PD with L-DOPA: mean ± SEM = 28.06 ± 2.861 vs 18.75 ± 2.110, paired *t-*test: *P* < 0.001). There were significant differences between the “PD with L-DOPA” and “PD without L-DOPA” conditions in “upper limb rigidity on the recorded side” (“PD without L-DOPA” vs “PD with L-DOPA”: 1.44 ± 0.203 vs 0.81 ± 0.855, *P* = 0.001) and “upper limb bradykinesia on the recorded side” (3.19 ± 0.458 vs 2.13 ± 0.507, *P* = 0.001), but not in “upper limb tremor on the recorded side” (1.69 ± 0.218 vs 1.75 ± 0.250, *P* = 0.751) (Fig. 2). The MMSE score was higher than 23 except for patient No 9. MoCA-J score was higher than 20 in all of the patients and was between 21 and 25 in nine patients [defined as PD-mild cognitive impairment (MCI)] [27,28] (Table 1).

### 3.2 LTP-like plasticity induced by QPS

**Fig. 3:**
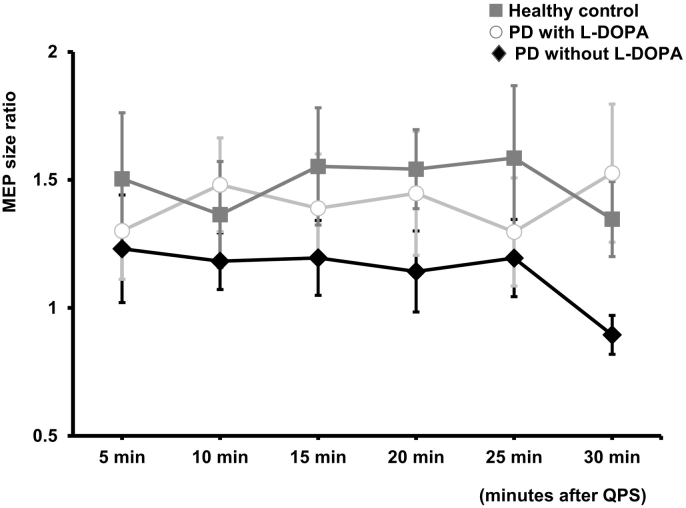
Time courses of mean MEP size ratio after QPS5. Diamonds (◆), circles (○) and squares (■) represent “PD without L-DOPA”, “PD with L-DOPA” and healthy controls (HC), respectively. The x-axis indicates time points after QPS5 and the y-axis MEP size ratio. Two-way repeated-measure ANOVA showed a significant effect of the factor “GROUP”, but not of the factor “TIME”. The post hoc analysis showed significant differences between “PD without L-DOPA” and “PD with L-DOPA” and between “PD without L-DOPA” and HC, but not between “PD with L-DOPA” and HC. Error bars are 1 SEM.

Neither RMT (“PD without L-DOPA” vs “PD with L-DOPA” vs HC: 44.88 ± 2.901 vs 44.38 ± 2.465 vs 45.38 ± 3.786 %MSO, *F* (2, 42) = 0.027, *P* = 0.973) nor AMT (“PD without L-DOPA” vs “PD with L-DOPA” vs HC: 32.56 ± 1.469 vs 32.81 ± 1.424 vs 32.39 ± 2.358 %MSO, *F* (2, 42) = 0.015, *P* = 0.985) were different between three groups. Fig. 3 shows the time courses of mean MEP size ratio after QPS5 under the conditions of “PD without L-DOPA” (diamonds), “PD with L-DOPA” (dots) and HC (squares). Two-way repeated-measure ANOVA showed a significant effect of the factor “GROUP” (*F* (2, 252) = 5.047, *P* = 0.007). The post hoc analysis showed significant differences between “PD without L-DOPA” and “PD with L-DOPA” (*p* = 0.042) and between “PD without L-DOPA” and HC (*p* = 0.010), but not between “PD with L-DOPA” and HC (*p* = 0.791). No significant difference was found in factor “TIME” (*F* (5, 252) = 0.159, *P* = 0.977) and interaction between “GROUP” and “TIME” (*F* (10, 252) = 0.379, *P* = 0.955).

These indicate that LTP was reduced in the early-stage PD patients and was restored to the normal level with L-DOPA.

### 3.3 Correlation between MEP size ratio and clinical measures

**Fig. 4:**
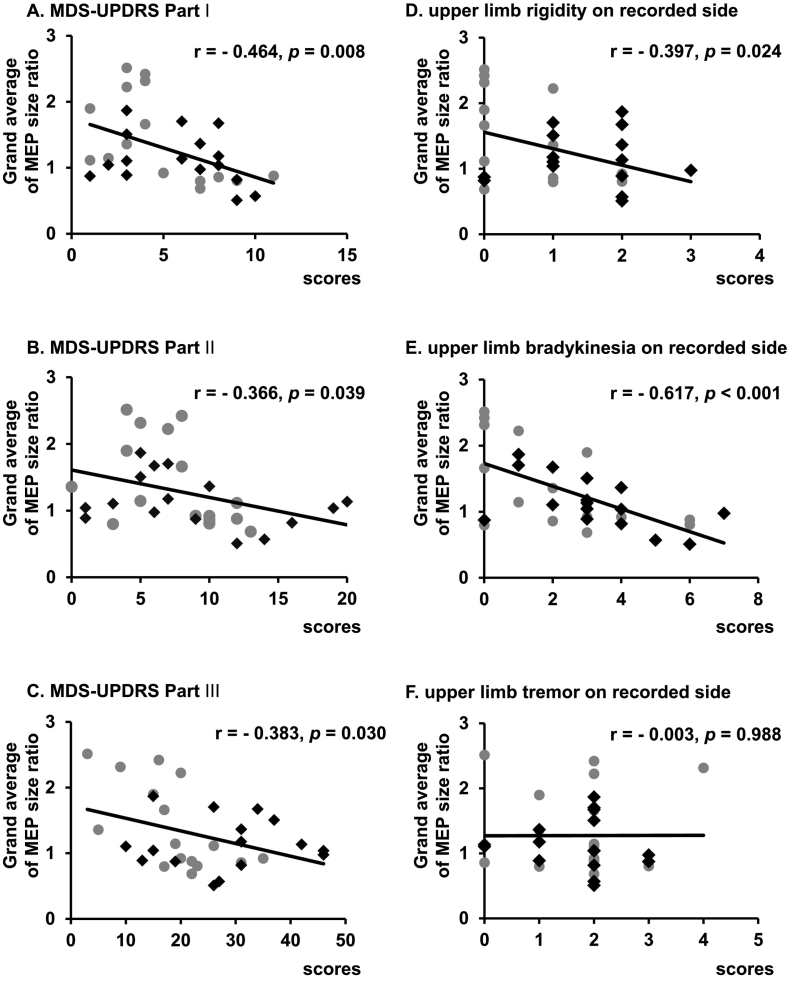
Correlations between the grand average of MEP size ratio after QPS5 and clinical scores. Diamonds (◆) represent “PD without L-DOPA” and grey dots (●) “PD with L-DOPA”. The x-axis in each diagram indicates scores of MDS-UPDRS Part I (A), MDS-UPDRS Part II (B), and MDS-UPDRS Part III (C), “upper limb rigidity on the recorded side” scores (D), “upper limb bradykinesia on the recorded side” scores (E), and “upper limb tremor on the recorded side” scores (F). The y-axes in all diagrams indicate the grand average of MEP size ratio after QPS5. MDS-UPDRS Part I and III score had significant negative correlations with the grand average of the MEP size ratio. In the sub-scores of MDS-UPDRS Part III, “upper limb rigidity on the recorded side” and “upper limb bradykinesia on the recorded side” scores showed significant negative correlations with the grand average of the MEP size ratios (D, E). In contrast, there was no significant correlation between “upper limb tremor on the recorded side” score and MEP size ratio (F).

Neither age, disease duration, L-DOPA dosage, MMSE score, nor MoCA-J score had a significant correlation with the grand average of the MEP size ratio (age: *r* = − 0.056, *P* = 0.761, disease duration: *r* = − 0.147, *P* = 0.421, L-DOPA dosage: *r* = − 0.218, *P* = 0.230, MMSE: *r* = 0.123, *P* = 0.503, MoCA-J: *r* = 0.340, *P* = 0.057). The MDS-UPDRS Part I, II, and III had a significant negative correlation with the grand average of the MEP size ratio (Fig. 4A, B, C). With regards to the MDS-UPDRS Part III subscores, “upper limb rigidity on the recorded side” and “upper limb bradykinesia on the recorded side” had significant negative correlations with the grand average of the MEP size ratio (Fig. 4D, E), whereas no significant correlation was observed with “upper limb tremor on the recorded side” (Fig. 4F).

## 4 Discussion

We showed two major findings in the present study; the LTP-like effect induced by QPS was restored by L-DOPA in PD patients, and the degree of LTP was negatively correlated with the severity of PD motor symptoms, especially upper limb bradykinesia and rigidity. The strong negative correlation with finger functional scores must be explained by the fact that the plasticity was evaluated for the hand muscle.

### 4.1 The motor cortical plasticity in PD patients and effects of L-DOPA

In PD patients, the LTP-like effect induced by QPS was smaller than HC and restored by L-DOPA. Several studies using PAS [4,6,10] reported the reduction of LTP in PD patients. However, the LTP after PAS was not enhanced by L-DOPA in drug naïve patients [13]. Studies using TBS did not show enhancement of LTP by L-DOPA in PD patients [7,30]. These differences from our results could be partly explained by the difference in the disease stages or in the medications between the studies. Most of the previous studies had recruited not only PD patients with L-DOPA monotherapy but also advanced PD patients taking multiple anti-parkinsonian drugs other than L-DOPA [4,7,10,30]. Here, we enrolled only PD patients with short disease duration [shorter than 7 years; 2.75 ± 0.49 (mean ± SEM)] who were drug-naïve or on L-DOPA monotherapy and who did not show any motor complications such as dopa-induced dyskinesia. This could be one possible explanation why we obtained results consistent with animal experimental data [3]. Another possible explanation may be different mechanisms underlying LTP induction between PAS, TBS, and QPS. PAS could be mediated by the spike timing-dependent plasticity involving both the sensory cortex and the M1 (heterotopic plasticity) [31], and TBS could be mediated by homotopic plasticity in the M1 but also be affected by the balance between intracortical facilitation and inhibition [32,33]. In contrast, QPS induces homotopic plasticity and does not induce any changes in the M1 inhibitory circuit [16]. This simplicity of the QPS mechanism may explain the consistency between our results and animal experimental results.

### 4.2 Motor symptoms and M1 plasticity

The most noticeable finding of this study was that parkinsonian symptoms (MDS-UPDRS I, II and III), especially upper limb bradykinesia and rigidity, correlated with the degrees of QPS-induced LTP.

Previous studies reported inconsistent results on the relationships between clinical symptoms and cortical plasticity in PD. Namely, no correlation at both the on and off states [10] or negative correlation at the off state [4]. In one study of drug-naïve PD patients [12], the PAS-induced LTP measured in a certain hand muscle negatively correlated with motor symptoms on the same side as the target muscle. This finding is consistent with the present result where the QPS-induced LTP-like plasticity had a strong correlation with symptoms on the side of target FDI. In another report [34], PAS-induced LTP had a negative correlation with bradykinesia in kinematic recordings of finger tapping, but not with UPDRS Part III score. In contrast, one study [13] demonstrated a positive correlation in drug-naïve PD patients, where the higher the degree of plasticity, the more severe the bradykinesia.

Here, we showed that the degree of LTP-induction negatively correlated with clinical motor scores. We also revealed a stronger negative correlation between the amount of LTP induction in the hand motor cortex and finger dysfunction, such as bradykinesia and rigidity. In this study, the comparison between the degree of plasticity and clinical measures at the same anatomical site (FDI and upper limb scores at the side of FDI) showed a strong correlation. In MPTP-induced PD model monkeys, abnormal firing of M1 neurons during some movements were found to contribute to the generation of bradykinesia [35,36]. In humans, the M1 has also been presumed to play an important role in the generation of bradykinesia [12,13,34]. Our results suggest that the LTP induction at the M1 has some relation to the mechanisms of bradykinesia generation, at least in the hand muscles.

Concerning rigidity, the relationship with cortical activities has been debated [37]. In a functional magnetic resonance imaging study [38], widespread cortical/subcortical connectivity, including the M1, was related with rigidity. However, another report suggested that rigidity was associated with spinal cord dysfunction [39,40]. Our results that bradykinesia and rigidity strongly related with motor cortical plasticity are consistent with those previous findings. The increased LTP in the M1 induced by L-DOPA could explain the improvement of these symptoms through L-DOPA intake. One possible hypothesis is that the LTP of the M1 somehow relates to the generation of rigidity and bradykinesia through the cortico-basal ganglia network.

A second possibility is that the amount of LTP in the M1 reflects the degree of D1 receptor activation in the M1. In human studies, the D1 receptor was predominant in the M1 [41,42], and D1 receptor activation contributes to LTP in the M1 [19]. Dopaminergic neurons in the ventral tegmental area (VTA) are known to project to the M1, and animal studies reported that VTA-to-M1 dopaminergic neurons contribute to motor learning and poor sequential movements [43,44]. VTA degeneration was shown to be associated with non-motor symptoms including sleep disturbance, apathy, and depression or anxiety in PD patients [45]. In our data, the amount of LTP induction also correlated with the non-motor scores of MDS-UPDRS Part I and II. It is plausible that LTP of the M1 is regulated by dopamine from the VTA. However, the details of the role of the VTA in PD have not been clarified. The motor cortical plasticity shown here may reflect the VTA-M1 dopaminergic projection function.

### 4.3 Limitations

In this study, when patients showed severe resting tremor, we could not study LTP on the more affected side. The lack of correlation with tremor score in this study may be explained by the lack of large variety of tremor score because patients with severe tremor were exclude from our study. Another limitation is that we did not investigate the less affected side. Comparison between the more and less affected sides in the same patients may give more information about the pathophysiological meanings of the plasticity. These points may be future study projects.

We studied only early-stage PD patients who took no medication or only L-DOPA. Therefore, we could not assess how the LTP-like effect induced by QPS relates to symptoms in the advanced stage. We studied both drug-naïve and patients chronically treated with L-Dopa. Studies of several different groups of PD patients may give us new information about the plasticity in PD. This is not the scope of the present study and will be a future project. In addition, we defined without L-dopa condition as after overnight withdrawal of L-DOPA treatment (at least 16 h) in patients already under L-DOPA medication considering the tolerance level of PD symptoms induced by L-DOPA reduction. It could not be long enough to completely exclude a long-lasting L-DOPA effect. However, because the PD symptoms were aggravated by this procedure in all the patients, we considered this comparison to be appropriate for the comparison between on and off states. Moreover, the effects of many other anti-parkinsonian drugs on LTP after QPS have not been studied. Further studies are needed in the future.

Even with these limitations, we propose that QPS5-induced motor cortical LTP may be a good biomarker of early-stage PD.

## 5 Conclusion

We showed that L-DOPA improved the QPS-induced LTP in PD patients, and parkinsonian symptoms such as bradykinesia and rigidity were negatively correlated with QPS-induced LTP. Our findings indicated that PD patients have cortical plasticity reduction which is restored by L-DOPA. Reduction in the motor cortical plasticity may produce the rigidity and bradykinesia in the studied hand muscles. LTP induced by QPS5 could be a good tool for estimating parkinsonian involvement objectively in PD patients.

## Declaration of Compeitng Interest

None.

## Author contributions

Shotaro Moriyasu: Design of the study, and acquisition, analysis and interpretation of data; Execution of the statistical analysis; writing of the first draft.

Takahiro Shimizu: Design of the study, and acquisition, analysis and interpretation of data; Execution of the statistical analysis; review and critique.

Makoto Honda: Acquisition of data; review and critique.

Yoshikazu Ugawa: Discussion about the interpretation of data; review and critique.

Ritsuko Hanajima: Conception and design of the study; Review and critique.

All authors have final approval of the submission.

## Funding sources

This work was supported in part by research grants from the Research Project Grant-in-aid for Scientific Research from the Ministry of Education, Culture, Sports, Science and Technology [grant numbers: 17K09809, 20K07866 (RH), (TS)and (YU)]. The study was also supported by a Grant-in-Aid for the Research Committee for Ataxic Diseases (RH) and Grants-in Aid from the Research Committee of CNS Degenerative Diseases, Research on Policy Planning and Evaluation for Rare and Intractable Diseases, Health, Labour and Welfare Sciences Research Grants (RH) from the 10.13039/501100003478Ministry of Health, Labour and Welfare, Japan.
